# Hotspots, Frontiers, and Emerging Trends of Superabsorbent Polymer Research: A Comprehensive Review

**DOI:** 10.3389/fchem.2021.688127

**Published:** 2021-07-29

**Authors:** Minmin Yang, Jihuai Wu, Geoffrey M. Graham, Jianming Lin, Miaoliang Huang

**Affiliations:** ^1^ College of Foreign Languages, International School, Huaqiao Univ., Quanzhou, China; ^2^ Engineering Research Centre of Environment-Friendly Functional Materials, Ministry of Education Institute of Materials Physical Chemistry, Huaqiao University, Quanzhou, China

**Keywords:** superabsorbent polymers, functional polymers, hydrogels, clay-based polymer composites, water absorbency

## Abstract

Superabsorbent polymer (SAP) is a kind of functional macromolecule with super-high water absorption and retention properties, which attracts extensive research and has wide application, especially in the areas of hygiene and agriculture. With reference to the Web of Science database, the SAP research literature from 2000 to 2019 is reviewed both quantitatively and qualitatively. By examining research hotspots, top research clusters, the most influential works, the representative frontier literature, and key emerging research trends, a visual panorama of the continuously and significantly increasing SAP research over the past 2 decades was presented, and issues behind the sharp increase in the literature were discovered. The findings are as follows. The top ten keywords/hotspots headed by *hydrogel* highlight the academic attention on SAP properties and composites. The top ten research themes headed by *clay-based composites* which boast the longest duration and the strongest impact have revealed the academic preference for application rather than theoretical study. Academically influential scholars and research studies have been acknowledged, and the Wu group was at the forefront of the research; however, more statistically significant works have been less detected in the last 10 years despite the sharper increase in publications. *Hydrogel*, *internal curing*, and *aerogel* are both current advances and future directions.

## Introduction

With the implementation of the United Nations Sustainable Development Agenda (The United Nations, 2015) and the improvement of the public awareness of health and environmental friendliness, the research, development, and applications of water superabsorbents have attracted increasing attention. Superabsorbent polymers (SAPs) or superabsorbents are one kind of functional macromolecule with hydrophilic polyhydroxy and slightly cross-linked three-dimensional (3D) network structures. Thanks to their unique structure and group features, SAPs have a super-high capability to absorb and retain water, being capable of absorbing water up to 4,000 times their own weight, with the absorbed water either not releasing or only releasing slowly under normal conditions, thus endowing this polymer material with special functions (Wu et al., 2000; Wu et al., 2005; Zohuriaan-Mehr and Kabiri, 2008).

In 1938, the first superabsorbent polymer was synthesized by thermally polymerizing acrylic acid (AA) and divinylbenzene in an aqueous medium (Buchholz and Graham, 1998). In the late 1950s, the hydrolysates of starch graft acrylonitrile (HSPAN) were synthesized by Russell et al. in the Northern Regional Research Lab of the United States Department of Agriculture. The products were mainly used in agriculture and horticulture as a hydrogel for plant growth and transportation (Mathur et al., 1996). In 1966, Fanta et al. grafted acrylonitrile onto wheat starch and obtained a superabsorbent product with a water absorption rate of 300–1,000 times its own weight and good water retention property (Fanta et al., 1966). The relative expensiveness and the inherent structural disadvantage (lack of adequate gel strength) are considered to be the main factors for its early market failure. The first commercial superabsorbent polymer was produced in 1970 by alkaline hydrolysis of starch-g-polyacrylonitrile (Buchholz and Peppas, 1994). The SAPs were industrially produced for hygienic application in the United States and Japan in the early 1980s (Brannon-Peppas and Harland, 1990), and the research, development, and application of SAPs then started to steadily grow.

The SAP products can be classified based on different aspects. According to the chemical composition of the monomer in the polymers, the SAPs can be classified as cross-linked polyacrylates or polyacrylamides and hydrolyzed starch-polyacrylonitrile (PAN) or cellulose-PAN graft copolymers (Wu et al., 2000; Zohuriaan-Mehr and Kabiri, 2008; Ullah, et al., 2015). Because polyacrylates and polyacrylamides are the most common SAP products, if it is not specified, the term “superabsorbent” usually refers to the copolymers of acrylic salt, either with acrylic acid (AA) or with acrylamide (AM). Based on the source of raw materials, the SAPs are categorized as synthetic (petrochemical-based) or natural. Natural sources include polysaccharides (such as starch, sodium alginate, and agarose) and polypeptides (such as collagen and gelatin) (Thakur et al., 2018). SAPs can be divided into four categories on the basis of the charge of the polymer repeat units: nonionic (neutral), ionic (anionic or cationic), ampholytic (both acidic and basic), and zwitterionic (both anionic and cationic). Mostly, commercial SAPs contain negative repeat units, so they are anionic.

**FIGURE 1:**
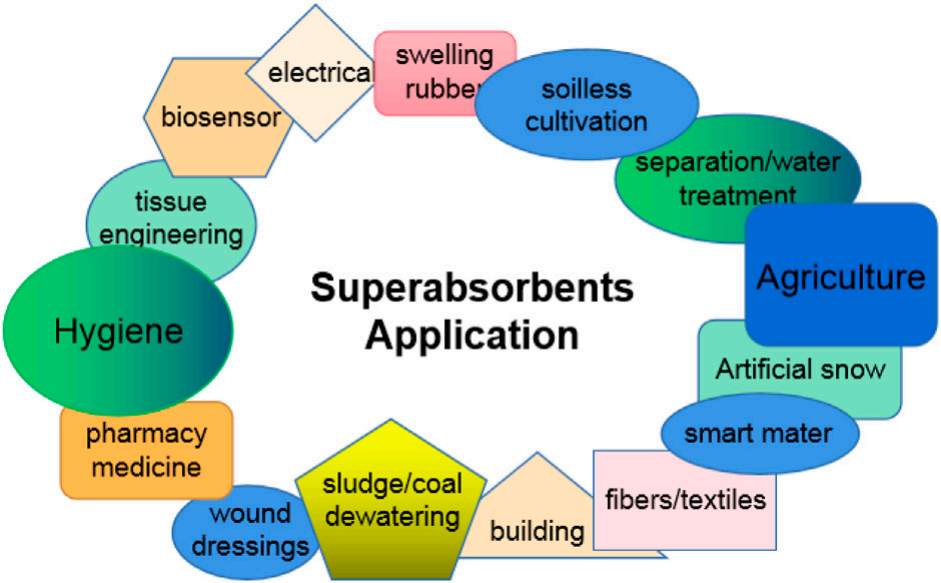
Applications of superabsorbent polymers.

Owing to excellent water absorbency, retention, and hydrogel properties, SAPs have obtained extensive applications (Wu et al., 2000; Zohuriaan-Mehr and Kabiri, 2008; Zohuriaan-Mehr et al., 2010; Ahmed, 2015), especially in fields such as hygienic products (Shahi et al., 2017a; Shahi et al., 2017b), agriculture (Zhang et al., 2014a; Zhang et al., 2014b; Guilherme et al., 2015; Fang et al., 2018), pharmacy and medicine (Mastropietro et al., 2012), tissue engineering (Stamatialis et al., 2008), wound dressings (Wiegand et al., 2011; Capanema et al., 2018), soil-less cultivation (Takeuchi, 2019), biosensors (Qasemi and Ghaemy, 2020), smart materials (Vosseler et al., 2012), electrical applications (Boruah et al., 2015), construction (Vieira et al., 2014), fibers and textiles (Majchrzycka et al., 2017), separation/water treatment (Zhou et al., 2016), sludge/coal dewatering (Zhang et al., 2017), and water-absorbing rubber (Adair et al., 2020). So far, the hygienic applications, particularly disposable diapers as described by Wu et al., 2000, have occupied the largest market share of SAP products and the agricultural applications the second largest. Figure 1 shows the application areas of SAPs.

## Methodology

### Data Collection

**FIGURE 2:**
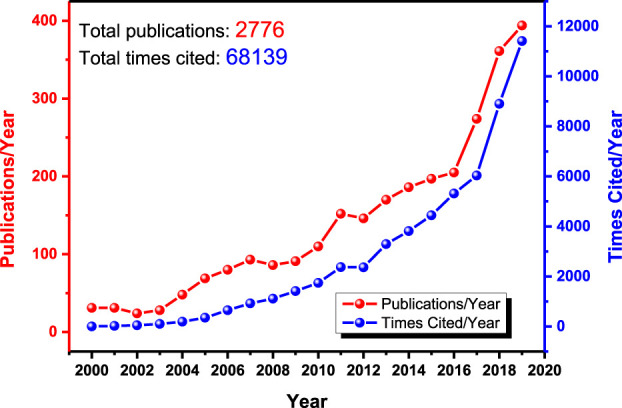
Publications and times cited per year with “superabsorbent” as a keyword, based on the Web of Science Core Collection.

In this review, research studies on superabsorbents from 2000 to 2019 are retrieved based on the Web of Science database, and the results are shown in Figure 2. The number of publications on SAPs per year steadily increases from 31 in 2000 to 383 in 2019, excepting a slow-down in the 2002 and 2003 bracket and two gently accelerated upward tides in 2007 and 2011. As shown in Figure 2, the citation of superabsorbent research maintains a slow and less obvious increase from 1999 to 2010, followed by a striking increase over the past 10 years; in this latter period, the numbers climb from about 2000 times in 2010 to an outstanding 10,000 plus in 2019. The past 20 years have witnessed a dramatic upswing in attention on SAP, and therefore this study singles out the period of 2000–2019 as the time span to examine, with the task of explaining the significant increase in the literature and targeting the research landscape to follow in the 21st century.

The above retrieval is performed by using “superabsorbent*” as the index word for a topic search. The wild card * is used in order to not miss the occasional plural usages of the topic word. This retrieval results in 2,559 bibliometric records with a document type of *Article*. Each record contains the metadata of a published article, including information of the author(s), article title, abstract, keyword list, and complete list of references cited by the article. These records are later exported to CiteSpace for further analysis. Adopting the complete reference lists as the primary objective of study is the primary feature of CiteSpace. As the literature on SAPs continues to grow rapidly, unless otherwise specified, the literature is that which was collected in March 2020.

### Data Processing

CiteSpace is a scientometric tool that mines, collects, processes, and visualizes academic text records (Chen, 2006), (Chen et al., 2012; Chen et al., 2014; Chen et al., 2018) (Chen and Song, 2019). In this article, the latest version, CiteSpace 5.6. R2, is used. To begin, the 2,559 retrieved records are fed into CiteSpace for data filtering and removal of duplicates. After that, a collection of 2,534 records is retrieved, providing the raw database for this study. After these procedures and calculations, the CiteSpace project will output three important results: a co-occurring keyword network, co-citation document networks, and citation burst detection. Adopting these three yields as the quantitative bases, the knowledge domains in the superabsorbent topic can be explored.

### Objectives

In this review, the increasing literature on SAP research published from 2000 to 2019 is examined. A visual panorama of research clusters and the landmark literature has been presented. Issues behind the sharp increase in research attention have been approached, namely, what are the prevailing research hotspots and clusters and what preferences do they suggest with regard to present studies, what works have been significant and pivotal in the development of SAP research, and what are the current advances and future directions of research.

## Research Hotspots

### Co-Occurrence Keyword Network

**FIGURE 3:**
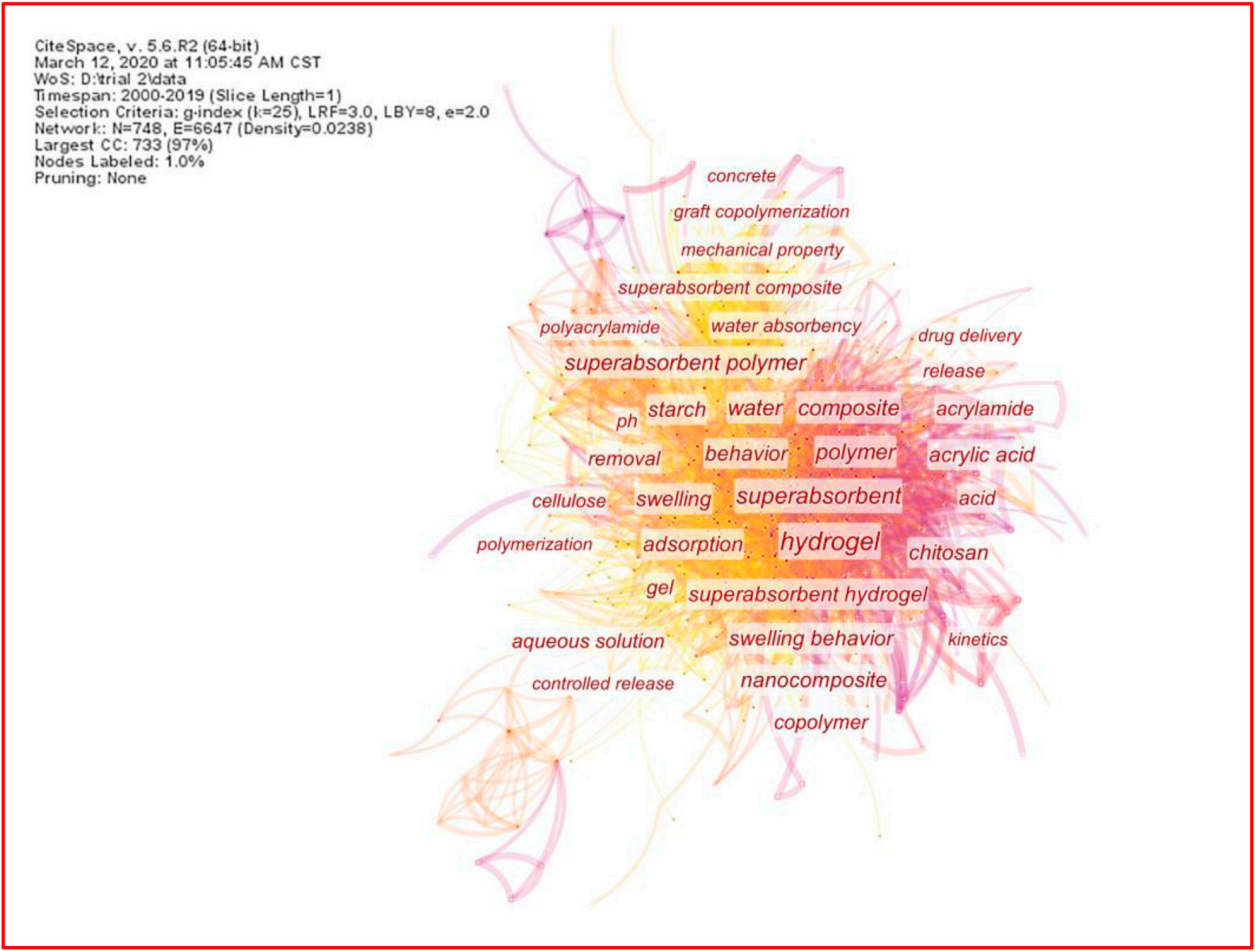
Co-occurring keyword network of superabsorbents (frequency >100) in 2000–2019 from CiteSpace.

Keywords are the labels indicating the significant concepts or core contents of an article. Co-occurring keywords are research hotspots and main topics of shared interest in the academic field (Xiao et al., 2017; Chen et al., 2012). The priority level, collaboration, and connection of the keywords in the SAP literature are detected using CiteSpace’s co-occurring keyword network (Xiang et al., 2017). A simplified co-occurring keyword network of superabsorbent research was obtained using the minimum spanning tree (MST) algorithm. The network includes 6,617 links and 748 nodes (words). Every node represents a keyword or keyword phrase, and the size of each node (or word) corresponds to the co-occurring frequencies of the keywords. The link between two nodes indicates the interconnectivity of the two keywords, and the stronger and clearer the link, the more frequently they co-occurred in the same batch of articles. Among them, 34 keywords with a cited frequency of >100 times are highlighted in Figure 3. Most of these SAP keywords are closely connected to each other, and therefore, it can be concluded that most studies in the field of SAPs are interlinked.

### Top Ten Research Hotspots

Table 1 shows the 34 co-occurring keywords with a higher co-occurrence frequency of up to more than 100 times. It can be seen that except for meta-words such as superabsorbent, superabsorbent polymer, polymer, water, and behavior, the highest co-occurring keyword is *hydrogel* (including *superabsorbent hydrogel* and *gel*). Hydrogel is the existing state in liquid and the main application of superabsorbents; thus, that is the keyword with the highest frequency. In Web of Science, nearly half of the articles relevant to superabsorbents have *hydrogel* as one of their keywords, which is consistent with the results of the CiteSpace analysis. *Composite* (including *nanocomposite* and *superabsorbent composite*) is another high-frequency co-occurring keyword in the SAP literature, attracting the most academic attention and forming the largest research cluster. *Swelling* (including *swelling behavior*) also appears as a high-frequency keyword. As is well known, SAPs boast two most distinctive properties: “high-absorbent” and “swelling.” The former, although a frequently appearing article keyword, is excluded from discussion for it is a meta-word of this study, and consequently, the latter alone makes it to the highest-frequency co-occurring keyword list.

**TABLE 1:** Research keywords in order of frequency in 2000–2019 (frequency >100).

Number	Frequency	Year	Keyword	Number	Frequency	Year	Keyword
1	971	2000	Hydrogel	18	197	2000	Gel
2	592	2000	Superabsorbent	19	195	2005	Aqueous solution
3	405	2000	Superabsorbent polymer	20	188	2000	Copolymer
4	388	2000	Polymer	21	179	2000	Acrylamide
5	379	2003	Composite	22	160	2000	Water absorbency
6	345	2000	Water	23	158	2005	Superabsorbent composite
7	316	2003	Superabsorbent hydrogel	24	152	2002	Release
8	312	2000	Swelling behavior	25	137	2001	pH
9	308	2001	Behavior	26	133	2008	Cellulose
10	285	2001	Adsorption	27	121	2000	Kinetics
11	270	2001	Swelling	28	119	2001	Controlled release
12	267	2004	Chitosan	29	119	2008	Mechanical property
13	257	2001	Starch	30	116	2005	Graft copolymerization
14	257	2004	Nanocomposite	31	111	2005	Polyacrylamide
15	250	2003	Acrylic acid	32	108	2001	Polymerization
16	222	2000	Acid	33	107	2004	Drug delivery
17	221	2009	Removal	34	102	2001	Concrete

Among the top items of Table 1, the keywords *composite*, *chitosan*, *starch*, *acrylic acid*, *copolymer*, *acrylamide*, and *cellulose* all fall under compositions of materials. Chitosan, starch, and cellulose are natural (polysaccharide- and polypeptide-based) raw materials of SAPs. Acrylic acid (AA) and acrylamide (AM) are two main synthetic (petrochemical-based) crude materials of SAPs, and acrylic acid and its salts (acrylates) are the most major components in SAP products, so it is not surprising that these words of SAP compositions justifiably become one string of hot keywords. The keywords *swelling*, *adsorption*, *pH*, *removal*, *release*, and *mechanical property* can all be categorized as properties of materials. SAP properties therefore stand out as another strong interest field of investigators.

In short, carefully observing the mean year of co-occurrence keywords, it is found that twelve keywords appeared in 2000, eight keywords occurred in 2001, and none appeared in the last 10 years. This may be attributed to the common knowledge that the co-occurring of keywords needs accumulation over time. This also reveals the fact that there have not been as many revolutionary breakthroughs in the past decade as the decade before.

Additionally, the top ten hotspots of SAP research are *hydrogel*, *composite*, *swelling*, *chitosan*, *starch*, *acrylic acid*, *removal*, *copolymer*, *acrylamide*, and *release*, among which *hydrogel* is the most prevalent. They indicate the preference of the research attention since these keywords obviously revolve around the properties and compositions of SAPs, which is consistent with and can reflect the research characteristics of materials science.

## Research Themes

### Co-Citation Document Network

**FIGURE 4:**
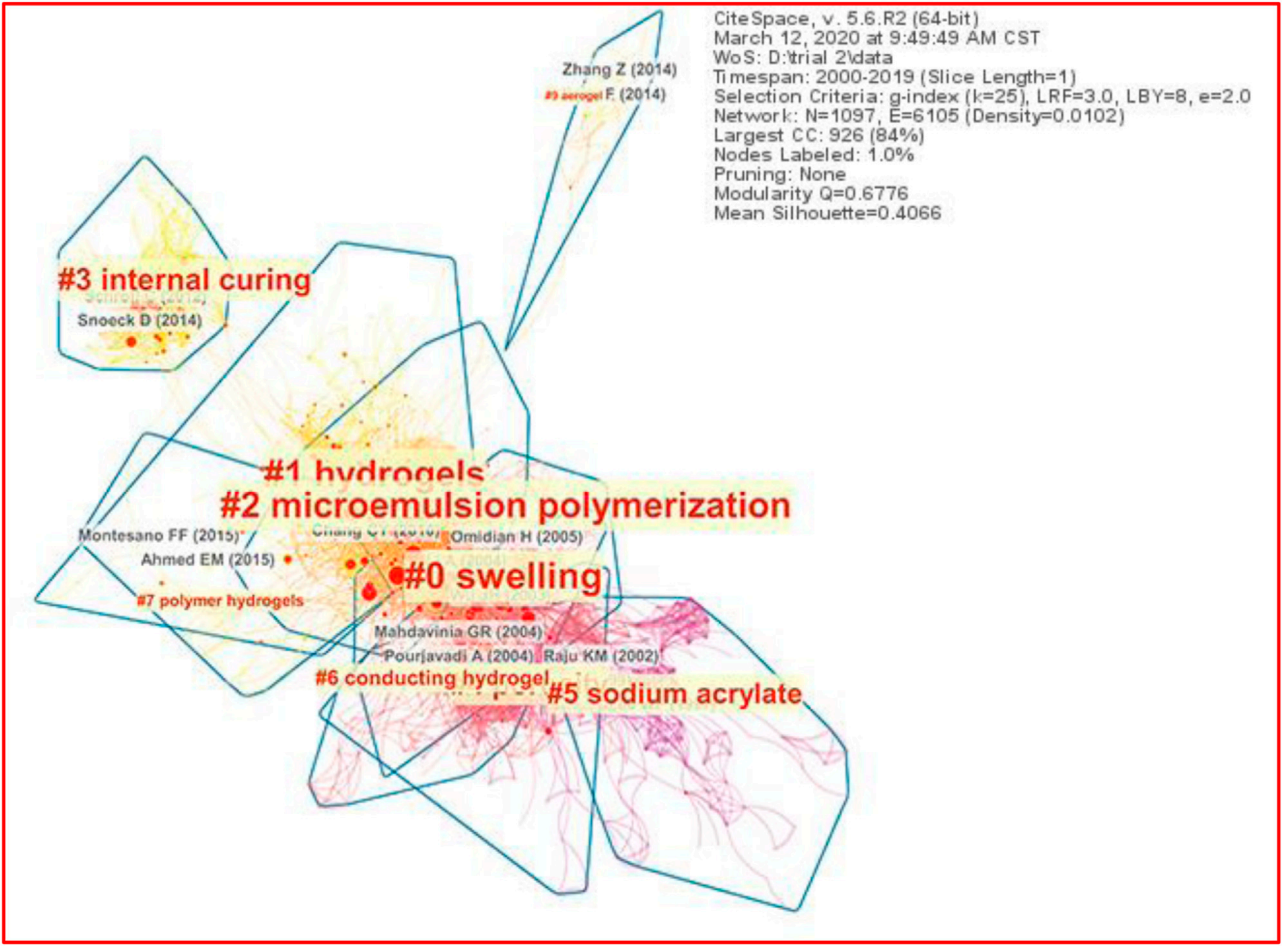
Co-citation document network (top 10 clusters) of superabsorbents in 2000–2019 from CiteSpace.

Each research article often cites several references. If two references are often cited together, it is obvious that there must be some association between the two references (Xiao et al., 2017). If there is such an association among multiple references, a document cluster can be established. A few document clusters of co-cited references weave a co-citation document network. The most prominent and unique feature of CiteSpace is the construction of a clustering and co-citation document network (Chen 2006). Figure 4 shows the co-citation document network of SAPs; the nodes stand for the references, and the line segment between the nodes represents the frequencies of them being cited by the same articles. The bigger the nodes, the more frequently they are co-cited. The nodes in the same cluster are tightly connected, while the nodes in different clusters are loosely connected (Chen et al., 2012). This whole landscape network is seen as the general intellectual structure (Liao et al., 2018).

In this review, the 47,446 references are collected from 2,534 metadata, generating a hierarchical co-citation document network. Based on the threshold setting and sampling criteria as described in section 2.2, this network yielded 1,097 unique nodes (documents), 6,105 links, and 97 clusters. After scrutinizing the clusters’ parameters, only the 10 biggest clusters are highlighted and labeled in Figure 4. The most sizable cluster is labeled Cluster #0, followed by #1 and so forth.

Foci are set on the ten larger main clusters. This is primarily because the last cluster (#10) has only 12 members/articles, implying that those un-included clusters are numerous but extremely small, both in size and in academic influence. Cluster #8 is invisible from the network as it does not meet the requirement of cluster aggregation.

### Ten Research Themes

Table 2 shows the 10 clusters with the largest document (coverage) quantity in the co-citation document network regarding superabsorbents. Each cluster represents a collection of a certain entity that is closely related based on the degree of co-citation. In other words, each cluster represents a research theme.

**TABLE 2:** Top ten clusters in the co-citation document network of superabsorbents.

Cluster	Size	Silhouette	Mean year	Label (LLR)	Main feature
#0	164	0.58	2005	Swelling	Clay-based composites
#1	159	0.697	2012	Hydrogels	Hydrogels
#2	155	0.665	2009	Microemulsion polymerization	Sensitive hydrogel
#3	110	0.992	2014	Internal curing	Internal curing
#4	109	0.891	2000	Porosity	Porosity
#5	99	0.939	1997	Sodium acrylate	Sodium acrylate
#6	52	0.996	2002	Conducting hydrogel	Conducting hydrogel
#7	31	0.988	2012	Polymer hydrogel	Soil and plants
#9	17	0.996	2013	Aerogel	Aerogel
#10	12	0.988	2011	Slow release fertilizer	Slow release fertilizer

To describe the main characteristics of a cluster, CiteSpace extracts noun phrases from the titles, abstracts, or keywords of the clustered articles to name/label the cluster (Chen, 2006; Chen and Leydesdorff, 2014; Li et al., 2017; Xiang et al., 2017; Xiao et al., 2017). However, owing to the intelligence limitation of computer software, the cluster labels do not always reflect the most salient and significant research features of the clusters. After carefully analyzing the retrieval results and referring to highly co-cited and frontier literature, a final column is added in Table 2 to indicate the main features of the cluster.

In general, the document co-citation clusters are ranked and evaluated in three ways: by size/coverage, by silhouette value, and by the mean year. In terms of size/coverage area, the biggest is Cluster #0, labeled as *swelling*, and the main feature is *Clay-based composite*. As mentioned above, “swelling” is also a high-frequency keyword in the SAP literature. As concluded previously, the main feature of Cluster #0 is identified as clay-based composites. *Composite*, denoting a material composed of two or more kinds of a single substance, is another prevalent keyword of high-frequency co-occurrence, ranking higher than *swelling*. A clay-based composite is a composite containing a clay component (Kabiri et al., 2011), and clay-based superabsorbent composites can not only reduce the processing cost and enhance the performance of products but also improve the processability and increase the accessional value of clay minerals. This has understandably attracted widespread research attention, and it has become the largest cluster in the SAP area (Wu et al., 2000). Prof. Jihuai Wu at Huaqiao University, China, was the first to report the research on the clay-based SAP composites, as early as 2000 (Wu et al., 2000).

The cluster with the second biggest coverage is Cluster #1, labeled *hydrogel* and mainly featuring *hydrogel*. Hydrogel is defined as a state of macromolecules which swells in water and retains a considerable portion (>20%) of water in the structure without being dissolved in water (Zohuriaan-Mehr and Kabiri, 2008). Hydrogel is, in many cases, just another literal expression of SAPs and is thus the most frequent co-occurring keyword and the second biggest cluster in the SAP literature. As a matter of fact, some hydrogel literature is also distributed in other clusters, such as the clusters labeled *Conducting hydrogel* (#6) and *Polymer hydrogel* (#7) and in Cluster #2, featuring *Sensitive hydrogel*. The Iran Polymer and Petrochemical Institute (IPPI) shows great commitment to SAP hydrogel studies, and they have established the Superabsorbent Hydrogel Division to this end. Prof. Zohuriaan-Mehr and colleagues have made an important contribution to the research on Cluster #2.

With respect to the silhouette value, the SAP literature’s ten biggest clusters all possess rather high silhouette values, which suggests and promises reliable and meaningful results of clustering (Xiao et al., 2017). Three clusters possess silhouette values almost as high as 1: Cluster #6 with 0.996, Cluster #9 with 0.996, and Cluster #3 with 0.992, as listed in Table 2. Interestingly, Cluster #9 (*Aerogel*) and Cluster #3 (*Internal Curing*) are distributed like islands isolated from the mainland of the SAPs shown in Figure 4. This distribution pattern can be ascribed to the fact that internal curing and aerogel are smaller branches of the SAP field, and research work on them can be carried out relatively independently.

As for the mean year of the cluster occurrence, Cluster #3 (*Internal Curing*) happened to be in the latest part of 2014, which is due to the emerging and speedy development of the construction industry in the preceding decade. The mean year of Cluster #9, labeled as *Aerogel*, was 2013, and this is a novel research frontier of SAPs. Cluster #5 (*Sodium Acrylate*) occurred in the earliest part of 1997, which is closely associated with the fact that sodium acrylate was the raw material of SAPs in this early stage and SAPs originated as a water-retaining agent for agriculture (Buchholz and Peppas, 1994; Wu et al., 2005).

A closer look at these label names and the prominent features of the top ten clusters can help clarify the intellectual structure of SAP study. Research on clay-based composites in Cluster #0 enhanced the properties of products and decreased the production cost of SAPs. Hydrogel of Cluster #1 is another expression of SAPs; aiming at smart materials, the sensitive hydrogel research of Cluster #2 focuses on the hydrogel’s responsiveness to temperature, acidity, ion concentration, and solvents. The use of SAPs in internal curing (Cluster #3) is a novel technology for concrete processing in the construction industry (Scrivener, et al., 2015). Porosity in Cluster #4 is related to SAP superporous hydrogels which can be used in drug-controlled release. Conducting hydrogels of Cluster #5 is related to the application of SAP hydrogels in the electrochemistry field (Wu et al., 2007; Lan et al., 2009). Sodium acrylate of Cluster #6 is a raw material traditionally used in the production of SAPs. Cluster #7 follows, concerning SAPs used in soil and plants, being the application of SAPs in agriculture. Cluster #9 concerns the preparation of aerogel by using SAPs, which is a novel technology and advanced material. Like Cluster #7 involves the application of SAPs in agriculture, Cluster #10, labeled *Slow Release Fertilizer*, centers on a smaller agricultural area in respect of improved fertilizer utilization (Azeem, et al., 2014; Campos, et al., 2015; Dai, and Huang, 2017; Chen, et al., 2018). In other words, the research characteristics of SAPs should be concluded as application study.

### High–Co-Citation Literature

In CiteSpace, the establishment of the co-citation document network is based on the cross-citation of the literature, with high–co-citation literature being the core and key of the co-citation network. The top ten co-cited articles in the SAP field are shown in Table 3.

**TABLE 3:** Top ten highest co-citation articles in the superabsorbent field.

Number	Cluster	Frequency	References
1	#2	139	Bao et al. (2011)
2	#0	112	Li et al. (2004)
3	#0	108	Wu et al. (2003a)
4	#0	103	Zohuriaan-Mehr and Kabiri (2008)
5	#0	101	Lin et al. (2001)
6	#3	100	Schrofl et al. (2012)
7	#2	98	Chang et al. (2010)
8	#6	85	Mahdavinia et al. (2004)
9	#2	85	Zhang et al. (2007)
10	#0	83	Kabiri et al. (2003)

Among all SAP literature, the most co-cited article is the article titled “Synthesis and swelling behaviors of sodium carboxymethyl cellulose-g-poly (AA-co-AM-co-AMPS)/MMT superabsorbent hydrogel,” written by Bao et al. (2011) at Shaanxi University of Science and Technology, China. In this study, a superabsorbent composite, acrylic acid (AA)/acrylamide (AM)/2-acrylamido-2-methyl-1- propanesulfonic acid (AMPS) on sodium carboxymethyl cellulose (CMC) and montmorillonite (MMT), was synthesized using the graft copolymerization method. The cellulose-g-P (AA-co-AM-co-AMPS)/MMT superabsorbent hydrogel comprised a porous cross-linked structure of MMT and CMC with side chains carrying carboxylate, carboxamide, and sulfate. These groups render the SAPs with excellent swelling property. The swelling of the synthesized superabsorbent is related to the particle size, pH, and concentration of external solutions. The relationship of the effects of four kinds of cationic salt solutions on swelling behavior of the superabsorbent hydrogels is: K^+^ > Na^+^ > Ca^2+^ > Mg^2+^. This article covers various important results concerning compositions, structure, and sensitive properties and therefore has universal referential significance, resulting in the article being the highest co-cited document in the SAP literature.

The literature with the second highest co-citation frequency of 112 was published by Li et al. (2004). A poly (acrylic acid)/attapulgite superabsorbent composite was synthesized using the graft copolymerization reaction. The synthesized SAP composite exhibited a good water retention under load and had a water absorption rate of 77 and 1,017 g·g^−1^ in 0.9 wt% NaCl solution and distilled water, respectively. This article is the first in a series of articles on attapulgite-based SAP composites. The excellent water absorption and retention properties are beneficial for agricultural and horticultural applications, providing recommendable and detailed synthesis and characterization methods for investigators.

In the top ten highest co-citation articles, there is only one article in Clusters #3 and #6, respectively, and the others are divided up by Clusters #0 and #2. The article in Cluster #6 was published by Mahdavinia et al. (2004) of SUT, Iran. In this article titled “Modified chitosan 4. Superabsorbent hydrogels from poly (acrylic acid-co-acrylamide) grafted chitosan with salt- and pH-responsiveness properties,” chitosan-g-poly (AA-co-AAm) hydrogel was synthesized and the reaction conditions were optimized. Water absorbencies of the hydrogels were discussed based on structural parameters, and the swelling behavior was explained according to the chemical structure and swelling theory. The synthesized hydrogels displayed cation exchange and salt-sensitivity properties. The pH switching and reversibility made the hydrogels intelligent polymers and good candidates for potential bioactive carriers.

It is worth noting that the meaning, frequency, and ranking of co-citation on CiteSpace are different from those on Web of Science. For instance, the article titled “Hydrogel: Preparation, characterization, and applications: A review.” by Ahmed (2015) has been cited 1,139 times and is the highest cited article in SAP research on Web of Science. However, the same article is co-cited 76 times in CiteSpace and is ranked the second highest co-cited article in Cluster #1. In this review article, hydrogel products are a class of polymer materials whose hydrophilic structure enables them to contain large amounts of water in three-dimensional networks. Hydrogel products are a kind of polymeric material. The hydrophilic groups and the lighted cross-linking structure make them contain large amounts of liquid in the three-dimensional network. Natural hydrogels were gradually substituted by synthetic types (petrochemical-based) owing to their advantages of high water absorbency, long serving lifetime, and wide raw material source. The classification, physicochemical properties, and technical feasibility of hydrogels are reviewed in this article. It also involves the technology used in hydrogel production, the meaning of the process design, the block diagram, and the optimization conditions of the preparation process.

Another example is the article by Korhonen et al. (2011) that has been cited 471 times and is ranked as the highest cited research article (non-review) on Web of Science. This article is co-cited only 11 times on CiteSpace and is ranked the third highest in Cluster #9.

## Research Frontier

### Landmark Works

Landmark works boast the strongest bursts of co-citations in scientometrics. “Burst” means a conspicuous abrupt change of some value in a relatively short period of time, and “Co-citation burst” is a rapid and extraordinary increase in citation counts; it marks a surge in citations of an article and is an indicator of the activity in the research area (Chen, 2006; Chen et al., 2014). Citation burst has two pronounced features: magnitude (strength of burst) and duration. Table 4 exhibits the top 30 strongest citation bursts of the literature on SAPs, ranked by their burst strength value, hence the Strength column in Table 4 is in bold. The year of publication of the article, the strength, the start year and the end year of the article citation bursts, and sundry other information are included. These are the landmark works that propelled the development of different schools of studies.

**TABLE 4:** Top 30 strongest citation burst articles in SAPs (ranked by Strength value, in bold).

References	Year	Strength	Begin	End	2000–2019
Lin et al. (2001), DOI	2001	**43.0603**	2003	2009	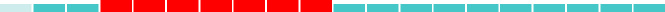
Wu et al. (2003b), DOI	2003	**37.1359**	2005	2011	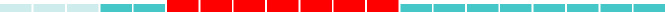
Li et al. (2004), DOI	2004	**33.5944**	2005	2012	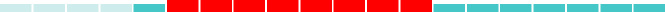
Wu et al. (2000), DOI	2000	**32.5811**	2001	2008	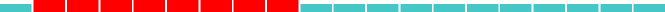
Zohuriaan-Mehr and Kabiri (2008)	2008	**29.4377**	2010	2016	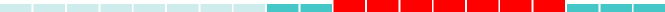
Kabiri et al. (2003), DOI	2003	**26.8232**	2004	2011	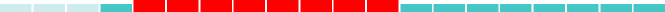
Mahdavinia et al. (2004), DOI	2004	**25.5905**	2006	2012	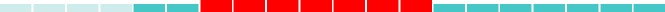
Omidian et al. (1999), DOI	1999	**24.8762**	2001	2007	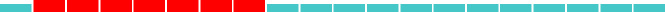
Zhang et al. (2007), DOI	2007	**24.3488**	2009	2015	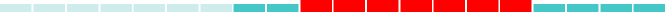
Schrofl et al. (2012), DOI	2012	**22.6569**	2015	2019	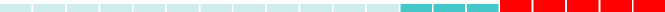
Omidian et al. (1998), DOI	1998	**22.0276**	2003	2006	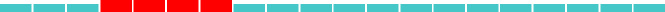
Lee and Yang (2004), DOI	2004	**21.8903**	2005	2011	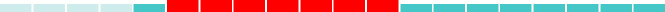
Guilherme et al. (2015), DOI	2015	**21.6236**	2016	2019	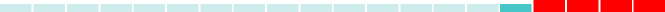
Chen and Zhao (2000), DOI	2000	**21.3162**	2003	2008	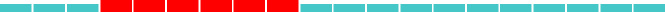
Justs et al. (2015), DOI	2015	**20.9553**	2017	2019	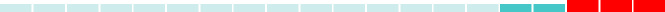
Liang et al. (2009), DOI	2009	**19.9043**	2012	2017	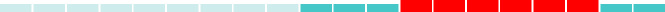
Chang et al. (2010), DOI	2010	**19.8301**	2013	2019	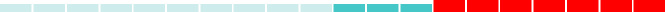
Li and Wang (2005), DOI	2005	**19.6339**	2006	2013	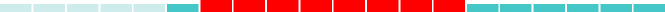
Snoeck et al. (2014a), DOI	2014	**19.4095**	2016	2019	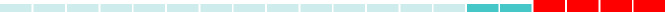
Snoeck et al. (2015), DOI	2015	**19.0338**	2017	2019	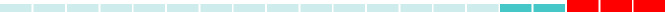
Snoeck et al. (2014b), DOI	2014	**18.7415**	2015	2019	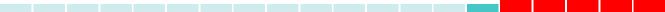
Zohuriaan-Mehr et al. (2010), DOI	2010	**18.2559**	2014	2019	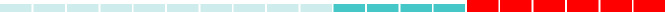
Bao et al. (2011), DOI	2011	**18.2194**	2013	2019	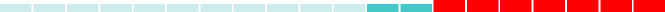
Pourjavadi et al. (2004), DOI	2004	**17.586**	2005	2012	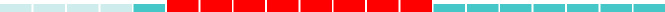
Zhang et al. (2005), DOI	2005	**17.1578**	2006	2010	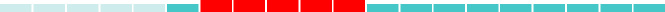
Kabiri et al. (2003), DOI	2003	**16.7428**	2004	2011	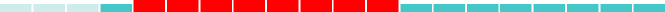
Li et al. (2007), DOI	2007	**16.738**	2008	2014	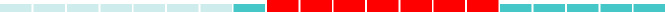
Kosemund et al. (2009), DOI	2009	**16.1938**	2011	2015	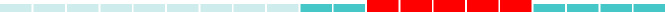
Mechtcherine et al. (2014), DOI	2014	**16.0454**	2015	2019	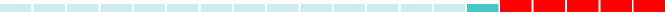
Lee and Chen (2005), DOI	2005	**15.2825**	2007	2013	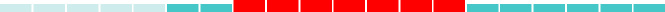

The top five articles with the longest duration time of 8 years in Table 4 are No. 4 by Wu et al. (2000), No. 6 by Kabiri and Zohuriaan-Mehr (2003), No. 18 by Li and Wang (2005), No. 24 by Pourjavadi A (2004), and No. 26 by Kabiri and Zohuriaan-Mehr (2003).

From Table 4, it may be observed that the citation bursts often began 1–3 years after the articles were published, which may be explained by the process of studying, understanding, and publishing up to the final citing of the article, which requires a certain period of time. Of the top 30 articles, 12 achieved citation burst 1 year after publication, another 12 articles took 2 years, four articles took 3 years, and the final two took 4 years and 5 years, respectively. Most articles in the top 30 (Table 4) have a shorter interval from publication to the beginning of their citation bursts than other documents, which should be attributed to their high quality and thus their rapid capture of international academic attention and influence.

Interestingly, the two articles mentioned above were both published by Omidian et al. 1998, 1999) in the Zohuriaan-Mehr group at the IPPI. In the article with the burst strength of 24.88 titled “Modified acrylic-based superabsorbent polymers (dependence on particle size and salinity)” (Omidian et al., 1999), SAPs were synthesized by inverse suspension and solution polymerization. The research results demonstrated that with the decrease in particle size, the absorption rate and the ultimate degree of absorption increased. A two-parameter Voigt model of the absorption rate was established: *ε(t) = σ*_*0*_ */E[*1−exp*{(t*_*0*_ *−t)/τ*_*0*_ *}].* Furthermore, the relationship between the ultimate degree of absorption and the cross-linker, as well as salinity, was subordinated to a power law: ultimate swelling = *k*{1/[cross-linker]}^*n*^; ultimate swelling = *k*{1/[salinity]}^*n*^.

The ultimate degree of absorption and the ultimate absorption rate are crucial for SAPs. In the article with the burst strength of 22.03 titled “A model for the swelling of superabsorbent polymers,” (Omidian et al, 1998) investigated the relationships between property data and preparation parameters. Plotting swelling against time showed mathematical relationships fitted to spring and dashpot models. By correlating the spring element with the expansion resistance of the superabsorbent, the dashpot element with the penetration resistance, and the element behavior with the polymerization system, the potential molecular factors affecting the absorption behavior could be identified. The relationship between the ultimate degree of swelling and the ratio of the cross-linker/monomer for different polymerization systems were established. These two classic works of theoretical research by Omidian et al. provide the academic basis for industrial production and practical application of SAPs and have become the only two items in the literature in the top 30 articles that were published in the last century, speaking to their exemplary impact on SAP research.

Notably, from Table 4, we see that only two articles with strong citation burst were published in the last century, about two-thirds of the top 30 were reported between 2000 and 2010, and about one-third were published in 2010–2020, which is basically consistent with the scientific research rule. However, it is regrettable that although the last decade has witnessed a much sharper increase in publications than the one before, the highest rank of an article published in the most recent decade is only 10th.

### Ten Pivotal Works

Similarly, the ten pivotal works are those that boast the top 10 highest burst values. Table 5 displays ten articles with the strongest citation bursts in the ten biggest clusters. The article with the strongest citation burst value of 43.06 was reported by Lin et al. (2001) of the Wu group at Huaqiao University. In this article, a novel poly (acrylic acid)/mica SAP composite with a water absorption of over 1,100 g·g^−1^ was synthesized by using the graft polymerization reaction between ultrafine mica mineral powder and partially neutralized acrylic acid. The article discussed the influence of the cross-linker, mica amounts, and the degree of neutralization on the absorbent properties, being one of the earliest reports on clay-based SAP composites. More importantly, a cross-linking structure model in which the ultrafine mica powder served as a supplementary network point of SAP composites was first proposed in this article. The significance of clay-based composites, a simple synthesis process, excellent performance, and the proposed structural model have attracted extensive attention and citation, making this report a typical strong citation burst report among all SAP documents.

**TABLE 5:** Articles with the strongest citation burst in the ten biggest clusters, respectively.

Cluster	Burst	References
#0	43.06	Lin et al. (2001)
#1	21.62	Guilherme et al. (2015)
#2	24.35	Zhang et al. (2007)
#3	22.66	Schrofl et al. (2012)
#4	24.88	Omidian et al. (1999)
#5	13.9	Lee and Wu (1997)
#6	25.59	Mahdavinia et al. (2004)
#7	8.54	Yang et al. (2014)
#9	7.27	Jiang and Hsieh (2014)
#10	11.83	Raju and Raju (2001)

Another strong citation burst article titled “Superabsorbent polymer materials: A review” possesses a prominent co-citation burst of 29.44, which was reported by Zohuriaan-Mehr and Kabiri (2008) at the IPPI. The IPPI attached great importance to SAP research (till date, 53 SAP articles have been published), particularly with regard to the SAP hydrogel area and the application in hygienic and bio-related areas. In this review, they introduced the background, types, structures, properties, methods, application, and research works. The major internal (synthetic) and external (environmental) factors affecting the SAP properties were demonstrated. The measurement methods of the important SAP properties, that is, swelling rate, absorption capacity (both under load and load-free), wicking capacity, swollen gel strength, residual monomer, sol fraction, and ionic sensitivity, were all scrutinized. The related disciplines of SAP applications, particularly the hygienic and agricultural fields, were also reviewed. Finally, the environmental and safety issues concerning SAP were also discussed. The high-quality and systematic introduction of this review provides momentous references for SAP research.

The article with the highest co-citation and the strongest citation burst in Cluster #3 was reported by Schrofl et al. (2012) at Technische Universität Dresden, Germany. They studied the relationship between the molecular structure and the efficiency of SAP as an admixture for high-strength concrete to alleviate autogenous shrinkage. The influence of SAPs’ two main characteristics of anionicity and degree of cross-linking was examined. These parameters are related to the kinetics of solution shrinkage and the release efficiency of cement. Internal curing has no adverse influence on the compressive strength of mortar. Systematic and objective research made this article highly praised.

(Lee and Wu, 1997) in Tatung University, Taiwan prepared a series of xerogels using 3-dimethyl (methacryloyloxyethyl) ammonium propane sulfonate (DMAPS), N,N′-methylene-bis-acrylamide (NMBA), and sodium acrylate (SA) as components by inverse suspension polymerization. The water absorbency decreased with an increase in the ionic strength of the salt solution. The pH effect and the thermal effect on the water absorbency for these xerogels were also inspected. This is an earlier study of SAP aerogels and also a study of sodium acrylate–based SAPs, therefore enjoying the strongest citation burst in Cluster #5.

### Representative Frontier Works

The research frontier is a research topic and field with the latest, advanced, and potential development characteristics (Chen, 2009). In CiteSpace, the representative frontier literature is those that have high citing frequency to the highly co-cited literature (Chen, 2010), the former being the focus of researchers. The ten representative frontier articles in the ten biggest clusters are listed in Table 6.

**TABLE 6:** Ten representative frontier works in the ten biggest clusters.

Cluster	Feature	References
#0	Clay-based composites	Wang and Wang (2010a), Wang and Wang (2010b)
#1	Hydrogels	Zheng and Wang (2015)
#2	Sensitive hydrogel	Wang et al. (2011)
#3	Internal curing	Farzanian and Ghahremaniezhad (2017)
#4	Porosity	Kabiri et al. (2011)
#5	Sodium acrylate	Chen and Zhao (2000)
#6	Conducting hydrogel	Tang et al. (2008)
#7	Soil and plants	Dehkordi (2018)
#9	Aerogel	Yang and Cranston (2014)
#10	Slow release fertilizer	Zhan et al. (2004)

The most representative frontier article in Cluster #0 was published by Aiqin Wang et al. (2009) at the Lanzhou Institute of Chemical Physics (LICP), Chinese Academy of Sciences (CAS), China. Having published more than a hundred SAP articles, being the most productive institute when it comes to SAP publications, the LICP, CAS, has made a great contribution to the field. In this article, a few SAP nanocomposites were synthesized by radical polymerization of attapulgite (APT), partially neutralized acrylic acid (NaA), and sodium carboxymethyl cellulose (CMC). The research results indicated that NaA was grafted on the CMC backbone, and APT was dispersed in the CMC-*g*-PNaA matrix and participated in polymerization. The superabsorbent nanocomposite possessed obvious pH sensitivity and time-dependent swelling property. The preparation of SAPs by grafting vinyl monomers onto natural polysaccharides and then compounding with inorganic clays was a preferred way owing to its environmental and commercial merits, rendering this article a most representative frontier article of the cluster of clay-based composites.

The most representative frontier literature in Cluster #1 was also reported by Zheng and Wang (2015) at the Lanzhou Institute, CAS. In this review with the title of “Superabsorbent with three-dimensional networks: From bulk hydrogel to granular hydrogel,” hydrogel is a three-dimensional network of polymer chains linked by physical or chemical bonds. The superabsorbent hydrogels show a fast adsorption rate, super-high adsorption capacities, wide pH independence, easy regeneration, and reusable ability, and the hydrogels are given the appellation “superabsorbent.” By contrast, the limitations of traditional bulk hydrogel make granular hydrogel look more attractive. The novel synthesized hydrogel adsorbents since 2000 were listed, and the main progress of the hydrogels is reviewed. The simple synthetic method of hydrogel preparation provides a lot of convenience for commercial development. The comprehensive knowledge on SAP hydrogel makes the article the representative frontier literature in Cluster #1. In addition, the most representative frontier literature in Cluster #2 was also reported by (Wang et al. 2011) at CAS.

Kabiri and Zohuriaan-Mehr at the IPPI made a respectable contribution to studies of superabsorbent porous hydrogels and published a representative frontier article in Cluster #4 of *Porosity*: “Synthesis of fast-swelling superabsorbent hydrogels: effect of crosslinker type and concentration on porosity and absorption rate” (Kabiri et al., 2003). In this article, using acetone and sodium bicarbonate as porogens, a highly porous and fast-swelling superabsorbent hydrogel was synthesized by rapid solution polymerization. The higher the concentration of the cross-linking agent, the shorter the gelation time. The shortening of gelation time led to more porogen bubbles in the viscose reaction mixture and higher porosity of the product. Both the swelling-on-cross-linker and swelling-on-saline dependencies have shown a power law relationship. With the increase in the degree of cross-linking, the adsorption capacity decreased, the adsorption rate increased, and the sensitivity to salinity decreased. The multiple and significant conclusions in this article promoted the research and development of superabsorbent porous hydrogels and made the article the most representative frontier article in Cluster #4 of *Porosity*.

In the article titled “Relationship between water absorbency and reaction conditions in aqueous solution polymerization of polyacrylate superabsorbents” (Chen and Zhao, 2000) prepared polyacrylate superabsorbents in a polyethylene bag by *in situ* aqueous solution polymerization. The effects of bath temperature, initiator dosage, isopropanol dosage, initial monomer concentration, and cross-linker content (Cc) on water absorbency (Q) were examined. The decline of molecular weight in the radical polymerization led to the increase in the chain-end of the network, which reduced the cross-linking density and increased the water absorption of the SAPs. An empirical formula, *Q* = 2.45Cc^−0.6^, was established, and the validity of Flory’s swelling equation was verified. Acrylic acid and its salt (acrylate sodium) are the most important synthetic (petrochemical-based) crude materials, and thus, this quality article about acrylate had been widely cited and had become the representative frontier article in Cluster #5 of *Sodium Acrylate*.

Tang et al. (2008) in the Wu group at Huaqiao University reported “A multifunctional hydrogel with high conductivity, pH-responsive, thermo-responsive and release properties from polyacrylate/polyaniline hybrid.” In the article, PANI/PAC hybrid hydrogel with a conductivity of 2.33 mS/cm was synthesized using the two-step aqueous polymerization method. A conducting mechanism of charge carriers (protons) hopping along the PANI chain was proposed. The PANI/PAC hydrogel had excellent thermo- and pH-sensitive properties. There were two obvious water adsorption peaks at pH > 12 and pH 4∼6. The novel synthesis method and predominant multifunctions made it the representative frontier literature of the *Conducting Hydroge*ls cluster.

## Emerging Trends

An emerging trend in CiteSpace indicates a growing and developing new trend in a certain specialty. A burst value change reflects a sudden change in co-citation frequency within a specific duration. If an article has high burst strength within a time span, it is considered strongly responsible for representing emerging trends. CiteSpace is specifically facilitative at the detection of the change of the burst value and the emerging trends in the scientific literature. The emerging trends can be identified by analyzing the citation burst strength of the literature (Yu et al., 2017). CiteSpace characterizes the emerging trends and the burst change in terms of a variety of visual patterns.

**FIGURE 5:**
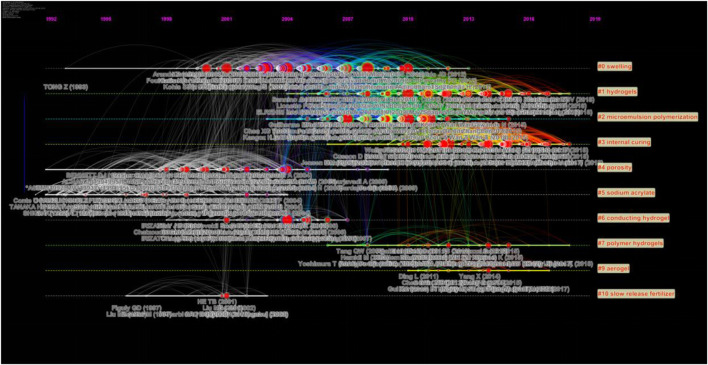
Timeline visualization of superabsorbent research of the ten major clusters from CiteSpace.

Figure 5 shows the timeline visualization of the ten major clusters of the co-citation network, and their evolution courses of all major clusters are vividly presented here. The nearest clusters to the right can be regarded as the most current research frontier, and only a few of them (#1, #3, and #9) continue to be exerting influence currently. They are therefore not out of date, namely, the “Emerging” trends, while others that are inactive presently are considered “Emerged” trends.

### Clay-Based Composites

Prominent on the timeline visualization are red circles that stand for the high–citation burst literature, which lead the directions of the evolution of the specialties and are critically decisive for the strength of the trends. Among all clusters, Cluster #0 has the most and the strongest citation burst literature; as a result, they bring about a research wave that spotlights the longest duration and the highest amplitude of burst.

Wu et al. (2000) at Huaqiao University, China, reported “Synthesis and properties of starch-graft-acrylamide/clay superabsorbent composite” in *Macromol Rapid Commun*. In this article, a graft copolymerization reaction involving acrylamide, potato starch, and clay mineral micro-powder was carried out to synthesize a starch-graft-polyacrylamide/clay superabsorbent composite, followed by a hydrolysis reaction with sodium hydroxide. The water absorbency of the clay-based composite achieved a record of 4,000 g H_2_O/g (this record has not yet been broken, so far). As a result, comprehensive absorption properties of the clay-based SAPs were enhanced and the production cost was reduced. The article is the first high-level report of clay-based SAP composites, in which a novel research field of SAPs was explored, represented, and highlighted by the first red circle on the timeline of clay-based composite SAP research (Cluster #0). The article achieves a citation burst as high as 32.58 and a persistent impact for 8 years.

**FIGURE 6:**
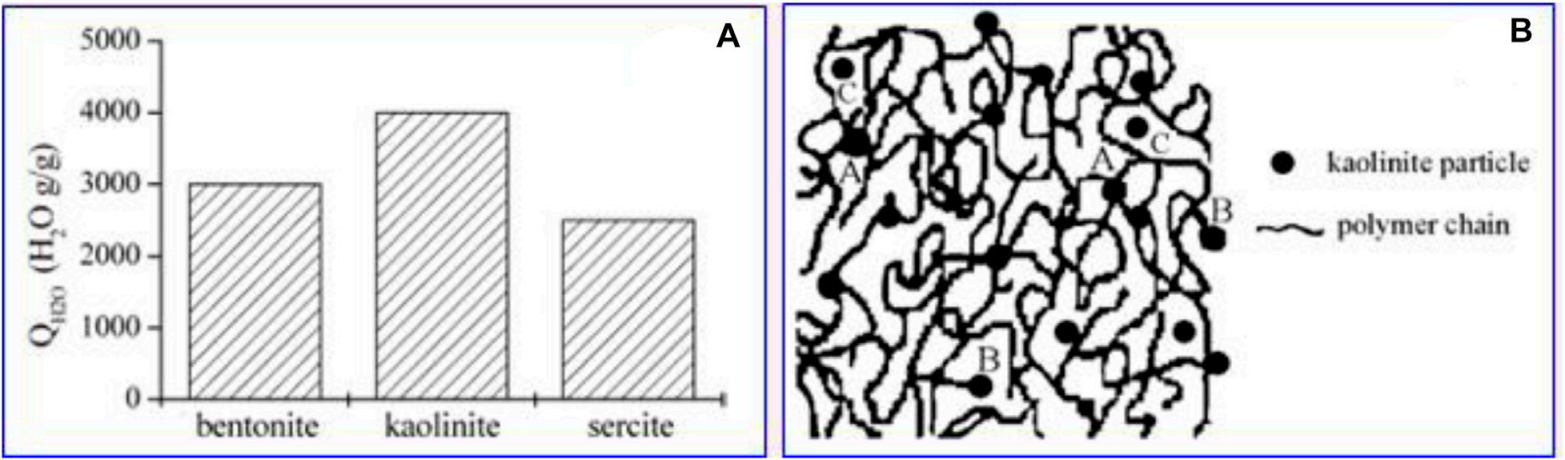
**(A)** Variation of Q_H2O_ of starch-graft-acrylamide/clay composites with bentonite, kaolinite, and sercite using clay (10 wt%) and potato starch (20 wt%). **(B)** Model for the cross-linkage structure. Reprinted with permission from Wu et al. (2003a). Copyright (2003) Elsevier Ltd.

The Wu group at Huaqiao University reported a second high-level research project in the same journal (Lin et al., 2001). In this article, a graft polymerization reaction between ultrafine mica mineral powder and partially neutralized acrylic acid was carried out to synthesize a poly (acrylic acid)/mica SAP composite. A cross-linking structure model of the poly (acrylic acid)/mica superabsorbent was first proposed (Figure 6). Probably owing more to acrylate-based SAPs than to acrylamide-based SAPs, on top of the advantages introduced by Wu et al. (2000), this article (Lin et al., 2001) obtains an even higher citation burst of 43.06 with a duration of 7 years, and this burst strength value is the highest among all SAP documents.

In 2003, the Wu group published a third article with a rather high citation burst of 37.24, ranking No. 2 among all SAP articles (Wu et al., 2003b). A graft copolymerization reaction involving mineral ultrafine powder, acrylamide, and potato starch was carried out to synthesize starch-graft-acrylamide/mineral powder composites, followed by an alkaline hydrolysis reaction with NaOH. The kaolinite-based composite possessed higher water absorbency than bentonite-based or sercite-based composites. By controlling the concentration of NaOH and the hydrolysis time in the saponification reaction, the type and quantity of hydrophilic groups can be changed. The collaborative absorption effect among the –COONa, –COOH, and –CONH_2_ groups was superior to that of the single –COONa, –COOH, or –CONH_2_ group. The polymerization reaction mechanism and the structure model of the clay-based composite were established. Wu’s group contributed a series of articles on the clay-based SAP composites (Wu et al., 2001; Wu et al., 2006), including the three highest co-cited articles in the top four of the strongest citation bursts list (Wu et al., 2000; Lin et al., 2001). It would therefore be fair to say that the Wu group has made a major noteworthy contribution to the establishment of clay-based SAP composite research so far.

The Wang group at the Lanzhou Institute, CAS also made a considerable contribution to the development of clay-based SAP composites. In the top 30 literature with strongest citation bursts, An Li, as first author, published three articles on clay-based SAP composites that boast burst values as high as 33.59, 19.63, and 16.74 in 2004, 2005, and 2007, respectively (Li et al., 2004; Li et al., 2007; Li et al., 2017). Their research focus is on the attapulgite-based SAP composites, and these exemplary works promoted the development of clay-based SAP composite research.

The IPPI was another important institute for clay-based SAP composite study. They mainly studied clay-based SAP composites from hydrogels. In the top 30 articles with strongest co-citation bursts, three reviews and one research article that have co-citation bursts as high as 29.44, 26.28, 18.26, and 16.74 were reported by Zohuriaan-Mehr MJ and Kabiri K at the IPPI from 2003 to 2010 (Kabiri et al., 2003; Zohuriaan-Mehr et al., 2010).

In 2012, an article titled “Nanocomposites based on poly (acrylamide-co-acrylate) and cellulose nanowhiskers” was published (Spagnol et al., 2012a), which is the last red circle on the timeline of Cluster #0. So far, clay-based SAP composite research had been developing for 13 years. Whether it can regain its brilliance remains a question for researchers to explore.

### Hydrogels

As mentioned before, hydrogels are the existing states in liquid and the main applications of superabsorbents, and hydrogel research is rather extensive and diverse, as shown by Cluster #2 of *Sensitive hydrogel* and Cluster #6 of *Conducting hydrogels*. Additionally, many high-level documents, such as the strong citation burst literature and frontier literature, are distributed in other clusters. For example (Kabiri et al, 2003) (Zohuriaan-Mehr et al, 2010), Cluster #0 also deal with hydrogels. It is therefore not easy to decide which article in which cluster should be listed as the first milestone article of hydrogel study.

Evident from Figure 5, SAP hydrogel research started in 2004 and has continued until now. A highlighted red circle was reported by Wang et al. (2009) at the Lanzhou Institute, CAS. They prepared a superabsorbent hydrogel composed of linear polyvinyl pyrrolidone (PVP) and sodium alginate-g-poly (sodium acrylate) (NaAlg-g-PNaA) with a pH-sensitive semi-interpenetrating polymer network (semi-IPN). The formation of the semi-IPN structure and the introduction of PVP greatly improved the water absorbency and swelling rate of the hydrogel. The synthesized hydrogel possessed excellent sensitivity to external pH stimulus. The semi-IPN hydrogel had an intriguing time-dependent swelling function in multivalent cationic saline solutions. This significant study achieves the highest co-citation frequency of 80 in Cluster 2#.

Ma et al. (2011) published the article titled “Synthesis and characterization of a novel super-absorbent based on wheat straw”; Chang and Zhang (2011) reviewed “Cellulose-based hydrogels: Present status and application prospects”; Spagnol et al. (2012b) described the study “Superabsorbent hydrogel composite made of cellulose nanofibrils and chitosan-graft-poly (acrylic acid)”; Zhou et al. (2013) conducted the project of “Superabsorbent nanocomposite hydrogels made of carboxylated cellulose nanofibrils and CMC-g-p (AA-co-AM)”; and Zhang et al. (2014a, 2014b) reported the study of “Synthesis, characterization, and swelling behaviors of salt-sensitive maize bran-poly (acrylic acid) superabsorbent hydrogel.” These articles have co-citation frequency counts as high as 39–56 and citation bursts from 9.83 to 12.23. They have sustained the booming of hydrogel study until now.

Guilherme et al. (2015) in Brazil published the review “Superabsorbent hydrogels based on polysaccharides for application in agriculture as soil conditioner and nutrient carrier.” The authors discussed the water transportation mechanisms from soil to plants based on mathematical models. Release of nutrients either from superabsorbent hydrogel (SH) or from granules coated by hydrophilic polymer was also discussed based on the often-used diffusion-based kinetic model and swelling-based kinetic model. This review guided the SH application in agriculture and obtained the strongest co-citation burst (21.62) in the *Hydrogels* cluster. In the same year, (Ahmed, 2015) in Egypt composed a review titled “Hydrogel: Preparation, characterization, and applications.” As mentioned in the title, this article gives a comprehensive introduction to the preparation, characterization, and applications of SHs, especially from the engineering perspective. This is the latest and most comprehensive overview of SHs and is cited extensively, driving it to be the most cited article (1,143 times) in the SAP field based on the Web of Science database.

Starting from 2004, the trend of hydrogel studies continues to be noticeably active. Even as recently as 2018, there was still a relevant article being published, achieving rather high co-citation burst (Kunal and Indranil, 2018). The SAP hydrogel research remains both an emerging trend and the most current developing SAP frontier, exerting strong influence continuously.

### Internal Curing

As the frontier research of SAP application, the emergence of internal curing is closely related to the development of the construction industry. The American Concrete Institute (ACI) defines internal curing as supplying water throughout a freshly placed cementitious mixture using reservoirs, *via* pre-wetted lightweight aggregates, which readily release water as needed for hydration or to replace moisture lost through evaporation or self-desiccation. SAPs possess excellent absorption and retention functions and are considered excellent admixtures for internal curing of cementitious materials. In 2007, the increasing application of SAP as a chief admixture of building material resulted in a report titled “Application of Superabsorbent Polymers in Concrete Construction (Technical Committee 225-SAP)” by the International Union of Laboratories and Experts in Construction Materials (RILEM). This state-of-the-art report summarized the available knowledge on SAP application in the construction field, providing a substantive foundation for SAP research and application in the building industry (Mechtcherine and Reinhardt, 2012).

Wang et al. (2009) investigated the water-release process of pre-wetted SAP particles in cement paste using a cracking viewer. Results indicated that incorporation of SAP obviously mitigated autogenous shrinkage and delayed internal relative humidity (IRH) decline at an early age, turning on the first red circle in the internal curing study (Figure 5).

Mechtcherine and Reinhardt (2012) at Technische Universitat Dresden, Germany, investigated the “Relation between the molecular structure and the efficiency of SAP as concrete admixture to mitigate autogenous shrinkage.” The SAPs with high anionic density quickly adsorbed the cement pore solution and subsequently steadily released. The cross-linking density had no obvious effect on the behavior of SAPs. SAPs counteracted the autogenous shrinkage of mortar, and the internal curing had no negative influence on the compressive strength of mortar. This is a representative document with the highest co-citation frequency of 100 and the strongest citation burst value of 22.66 in the cluster of *Internal Curing*.

De Belie and Snoeck et al. (2012) at Ghent University, Belgium, have published eight articles on SAP application with internal curing, including “Visualization of water penetration in cementitious materials with superabsorbent polymers by means of neutron radiography,” “Effect of high amounts of superabsorbent polymers and additional water on the workability, microstructure and strength of mortars with a water-to-cement ratio of 0.50” in 2014 (Snoeck et al., 2014a), “Self-healing cementitious materials by the combination of microfibers and superabsorbent polymers” in 2014 (Snoeck et al., 2014b), and “The influence of superabsorbent polymers on the autogenous shrinkage properties of cement pastes with supplementary cementitious materials” in Snoeck et al. (2015). These quality articles boast co-citation burst values as high as 15.24–19.41 and high co-citation frequency counts from 57 to 72, establishing and confirming the leading position and definite influence of the De Belie research group in internal curing research worldwide.

In 2015, Justs et al. (2015) at the Swiss Federal Laboratories for Materials Science and Technology (EMPA) reported “Internal curing by superabsorbent polymers in ultra-high-performance concrete.” In this article, the internal curing *via* SAPs was useful in reducing the autogenous shrinkage and the internal relative humidity. The SAP cavities were partially filled with portlandite during the cement hydration process. Although the mechanical properties were affected by SAP addition, a compressive strength of about 150 MPa could be reached in 28 days. This article obtained the second strongest co-citation burst value of 20.96 in Cluster #3 and promoted the development of SAP application in internal curing.

Since 2009, the evolution of internal curing research has been healthy and thriving. Until 2019 and 2020, there were still many articles being published (He et al., 2019; Snoeck et al., 2019; Shen et al., 2020), vouching for the fact that it is a veritable emerging trend and frontier field of SAP study.

### Aerogels

Aerogel is a synthetic ultralight porous material derived from a gel, in which the liquid component of the gel is replaced by gas (Kunal and Irdrani, 2018), different from the hydrogels, in which the 3D network of SAP is filled with liquid. Aerogel is a solid material composed of highly porous micropores and mesopores. When the density of aerogels is about 0.05 g·cm^−3^, the pores of aerogels usually account for more than 90% of the volume. The preparation of aerogels generally involves supercritical drying technology, that is, a sol–gel process to remove solvent from a gel (Johannes, 2014).

SAP aerogel research can be traced back to 2010, when an article titled “Carbon nanotube sponges” was reported by Gui et al. (2010) at Tsinghua University, China. Although the word “superabsorbent” did not appear in the article, many of the later studies on SAP aerogels are inspired by it. For this reason, the year 2010 is listed as the starting year of an emerging trend of SAP aerogel study.

**FIGURE 7:**
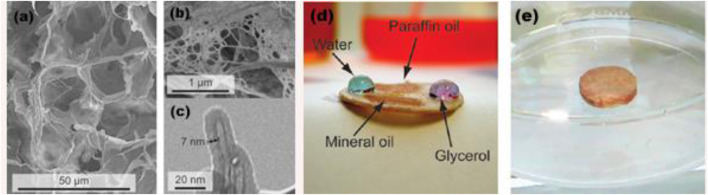
Microstructure of natural nanocellulose aerogels. **(A)** SEM image of freeze-dried nanocellulose aerogels, with fibrils packed into sheets and connected to form an open, porous aerogel structure. **(B)** Magnification of a nanoporous fibril sheet. **(C)** TEM image of a nanocellulose fibril coated with TiO_2_ (7 nm). **(D)** TiO_2_-coated aerogels are hydrophobic and oleophilic: the water and glycerol keep droplets (colored with Reactive Blue dye for clarity), whereas paraffin oil and mineral oil are readily absorbed. **(E)** Coated aerogel floating on water. Reprinted with permission from the Korhonen et al. (2011). Copyright (2011) American Chemical Society.

In 2011, Korhonen et al. (2011) in Finland carried out the study “Hydrophobic nanocellulose aerogels as floating, sustainable, reusable, and recyclable oil absorbents.” In the article, highly porous nanocellulose aerogels were prepared using the vacuum freeze-drying technique from micro-fibrillated cellulose hydrogels. Hydrophobic and oleophilic coatings (such as TiO_2_) were functionalized with the aerogels to obtain selective oil-absorbing materials floating on water (Figure 7). The surface-modified aerogel can be used to collect organic pollutants on the water surface. These materials can be reused after being washed, recycled, or incinerated with the absorbed oil, thus improving their application prospects in the environment. The facile preparation, excellent properties, and vivid figures and video of the article attracted extensive citation. Based on Web of Science, this article is the most cited SAP research literature (excluding reviews), boasting a cited frequency of 471 times (Korhonen et al., 2011).

Jiang and Hsieh (2014) at the University of California, Davis, United States, conducted research on “Amphiphilic superabsorbent cellulose nanofibril aerogels.” In this article, ultra-porous (99.9%) and ultralight (1.7 mg·cm^−3^) aerogels were prepared from cellulose nanofibrils (CNFs), and the CNFs were obtained by defibrillating from rice straw cellulose in 96.8% yield. The as-synthesized amphiphilic superabsorbent aerogels absorbed 210 and 375 times the water and chloroform, respectively, which were far superior to any previously reported cellulose aerogel. The hydrophobicity and lipophilicity of the amphiphilic aerogel was improved by the vapor phase deposition of triethoxy (octyl) silane on the aerogel. They can absorb 139–356 times the nonpolar hydrocarbons, polar aprotic solvents, and oils, 2–20 times higher than all previously reported polymers, cellulose, and carbonaceous aerogels. The excellent amphiphilic SAP aerogels have attracted the attention of academia, endowing the article with both the strongest co-citation burst and the highest co-citation frequency in SAP aerogel study.

The SAP aerogel study includes 40 articles. Among the top ten clusters, it is the second smallest and the latest to start. Being the newest emerging trend, it is still full of vitality.

## Summary

In summary, on the basis of the Web of Science database, the research patterns and historical development of the constantly and significantly increasing superabsorbent polymer studies are reviewed. The research hotspots were analyzed, and the most prevalent research keyword (research hotspot) is *hydrogel*. It is revealed that SAP compositions (i.e., composite, chitosan, starch, acrylic acid, copolymer, and acrylamide) and properties (i.e., swelling, removal, and release) constitute and dominate the top ten keywords. This prominent academic attention paid to SAP compositions and properties is in accordance with the research rule in materials science.

Mainstream research themes are detected, and the present intellectual structure is clarified. *Clay-based composites*, *Hydrogels*, *Sensitive hydrogels*, *Internal curing*, *Porosity*, *Sodium acrylate*, *Conducting hydrogels*, *Soil and plant*, *Aerogel*, and *Slow release fertilizer* research are identified as the ten major research clusters. It can be concluded that application study is the main feature in the SAP discipline.

Based on co-citation burst detection, the landmark literature that has been pivotal in the development of SAP research and the representative frontier literature of SAP study in the top ten clusters are highlighted. The highest co-cited literature on *Sensitive hydrogels* was reported by Ma and Bao; the highest co-citation burst literature on *Clay-based composite* was described by Lin and Wu at Huaqiao University; and the most representative frontier article on *Clay-based composite* was published by the Wang group at the LICP, CAS. The core contents and key significance of the pivotal literature are described. The most active and productive authors, key research institutions, and highly cited articles are also introduced.

Although the past 20 years have witnessed a sharp and constant increase in SAP studies, the relative lack of both research hotspots and pivotal works that are to promote the evolution of every school of study in the past decade, despite the especially sharp increase in publications, compared to the decade before, is a research gap significant enough to call for more attention and more creativity in the future of SAP developments.

In SAPs, the theme *Clay-based composite* has the most and the strongest citation burst documents, promoting a research trend with the longest duration and the highest amplitude; the Wu group from China was at its forefront.

Three active clusters of research are the prevailing and future directions of SAP development. Starting from 2004 until now, *hydrogel* research has been rather active and dispersed. It is still an emerging trend and the most current frontier of development. *Internal curing* research as the frontier of SAP application is related to the development of the construction industry; De Belie and Snoeck in Belgium contributed remarkably to it. The SAP *aerogel* study is the latest to start among the top ten clusters. It is the newest emerging trend and is still full of vitality.
